# Severe mental illness, common mental disorders, and neurodevelopmental conditions amongst 9088 lower court attendees in London, UK

**DOI:** 10.1186/s12888-022-04150-4

**Published:** 2022-08-12

**Authors:** Eddie Chaplin, Jane McCarthy, Salma Ali, Karina Marshall-Tate, Kiriakos Xenitidis, Denise Harvey, Jessica Childs, Samir Srivastava, Iain McKinnon, Louise Robinson, Clare S. Allely, Sally Hardy, Barry Tolchard, Andrew Forrester

**Affiliations:** 1 Institute of Health and Social Care, London South Bank University, London, UK; 2 University of Auckland, New Zealand & Visiting Senior Lecturer, King’s College London, London, UK; 3 South London and Maudsley NHS Foundation Trust, London, UK; 4 Together for Mental Wellbeing, London, UK; 5 Newcastle University, Newcastle upon Tyne, UK; 6 Manchester University, Manchester, UK; 7 Reader in Forensic Psychology at the University of Salford, Manchester, UK; 8 Affiliate member of the Gillberg Neuropsychiatry Centre at Gothenburg University, Gothenburg, Sweden; 9 Honorary Research Fellow in the College of Medical, Veterinary and Life Sciences affiliated to the Institute of Health and Wellbeing at the University of Glasgow, Glasgow, UK; 10 University of East Anglia, Norwich, UK; 11 Teesside University, Middlesbrough, UK; 12 Forensic Psychiatry, Cardiff University, Cardiff, UK

**Keywords:** Court liaison and diversion, Criminal Justice, Mental health, Screening, Mental disorder, Neurodevelopmental disorders, Alcohol and substance use

## Abstract

**Background:**

Court Mental Health Liaison and Diversion Services (CMHLDS) have developed in some countries as a response to the over-representation of mental illness and other vulnerabilities amongst defendants presenting to criminal justice (or correctional) systems. This study examined the characteristics and rates of mental disorder of 9088 defendants referred to CMHLDS.

**Method:**

The study analysed service level data, obtained from the National Health Service’s mental health data set, to examine characteristics relating to gender, ethnicity and comorbidity of common mental and neurodevelopmental disorders at five CMHLDS across London between September 2015 and April 2017.

**Results:**

The sample included 7186 males (79.1%) and 1719 females (18.9%), the gender of 183 (2%) were not recorded. Of those referred, 6616 (72.8%) presented with an identifiable mental disorder and 503 (5.5%) with a neurodevelopmental disorder (NDD). Significantly higher rates of schizophrenia were reported amongst Black defendants (*n* = 681; 37.2%) and Asian defendants (*n* = 315; 29%), while higher rates of depression were found amongst White defendants (*n* = 1007; 22.1%). Substance misuse was reported amongst 2813 defendants (31%), and alcohol misuse amongst 2111 (23.2%), with significantly high rates of substance and alcohol misuse amongst defendants presenting with schizophrenia or personality disorder.

**Conclusions:**

This is one of the largest studies to examine mental health needs and vulnerabilities amongst defendants presenting to CMHLDS. It will enable an improved understanding of the required service designs and resources required to manage the healthcare pathways for people attending CMHLDS.

## Background

Court Mental Health Liaison and Diversion services (CMHLDS) have been operating to support vulnerable people through the criminal justice (correctional) system (CJS) in England and Wales for at least 25 years [1]. These services operate principally within police stations and lower (Magistrates’) courts, and they assist people at whatever stage in the process they present. This includes before or after an arrest has taken place or charges have been laid, while attending court as an accused person, or following conviction [2]. The CMHLDS do not provide treatment but offer assessment and informed decision-making in regards to charging, sentencing and disposal of a defendant including referral or signposting to appropriate services [3]. There can be important operational differences between CMHLDS arising from different local interpretations of policy, legislation and local service pathways [4]. Operationally services have traditionally ceased following the end of police or court involvement. Healthcare services are increasingly being provided within the CJS as part of a joint pathway [5]. Referral pathways between criminal justice and health services are designed to map the range of available services to support the person, their relationship and how they work together. However, in many locations, there have been problems with sharing information across Criminal Justice Pathways [6] made worse by poor access to high-quality mental health services [7].

The main focus of CMHLDS is on people who present with major mental illness; however, their remit can include other vulnerable groups including people with Neurodevelopmental Disorders (NDD)s, substance and alcohol misuse or those at risk of self-harming and suicidal behaviour [1, 8]. To date, little research is available regarding the characteristics of court defendants referred to CMHLDS. Referrals are received from several routes including Forensic Mental Health Practitioners, probation (who will often provide initial screening), health agencies, Magistrates, court detention staff, police and voluntary services [1]. In England and Wales, key national reports have highlighted deficiencies in existing CMHLDS and described the inequalities experienced by people from groups that have not traditionally been prioritised within these services, including those with intellectual disability (ID) [9, 10], autism spectrum disorders (ASD) and attention deficit and hyperactivity disorder (ADHD) [2]. There are calls for CMHLDS to be extended to people with multiple vulnerabilities who have no single vulnerability that meets the eligibility criteria. A recent evaluation of CMHLDS in England highlights that of the received referrals, many defendants have more than one need, 71% had a mental health need, with 20% identified as having more than one mental health need and 52% identified with substance or alcohol misuse [11].

There has been a lack of research emphasis within CMHLDS when compared to other areas of the CJS such as prisons and police custody. It is important to consider each of these settings’ interdependence given that screened rates of psychiatric and NDDs can vary according to the setting and that people in the criminal justice system move between criminal justice and healthcare services. Comparing rates of mental illness (MI), community prevalence rates has been estimated at 3.4%, for depression, 3.8%, for anxiety disorders, 0.3% for schizophrenia, 1.4% for alcohol use disorder and 0.9% for drug use disorder [12, 13], with rates for a specific NDD estimated at between 0.8–1% for ASD, 0.5–2% for ADHD and 0.4–3% for ID [10, 14]. In comparison, a London-based study of 600 police detainees, found rates of psychiatric disorders at 39%, with 8% screening positive for psychotic disorders and 5–8% with major depression [15]. In an 18-month review of 1092 cases at two London police stations, 66.8% were reported to have psychiatric disorders. These included 20.1% with psychotic illnesses, 16.6% with depression, 21.2% with primary drugs or alcohol issues, and 6% with intellectual disability [16]. More recent work found that 40% met the threshold for a lifetime prevalence of major mental illness, with a screened prevalence of 14% for ADHD and 4% for ID [17].

The risk of psychiatric disorder is complex and there is evidence to show higher rates from those referred to CMHLDS arriving from custody, compared to community arrivals. A study of those appearing in court from overnight police custody found that they had a higher rate of psychiatric disorder compared to community court attendees (6·6% vs 1.3%) [8]. Of 99 defendants with psychiatric disorders, 66 (66.6%) had ‘serious’ conditions. Meanwhile, high levels of suicidality are reported amongst these samples [17, 18].

The current study aimed to analyse a database of over 9000 defendants to provide a comprehensive description of people with psychiatric and NDDs who are processed through the lower courts. We aimed to examine rates of these disorders and to determine whether differences arise regarding protected characteristics (e.g., age, gender and ethnicity) to determine whether they should be taken into greater consideration in future service developments [9, 19, 20].

## Method

We examined the mental health and related characteristics of 9088 defendants referred to CMHLDS across five London Magistrates^1^ between September 2015 – April 2017.

### Procedures

We obtained data from the National Health Service Minimum Data Set (MDS), which had been collected monthly during the study period. Data forms were sent to National Health Service England (NHSE) by providers every month and collated centrally into aggregated data. The data reflects the contents of current *clinical* and custody records and is obtained directly from front-line court and liaison and diversion service staff with the diagnostic data conforming to ICD-10 criteria. The data informing these records are compiled by several different disciplines including Forensic Mental Health Practitioners (FMHP), who assess, refer and ensure effective partnership working in the court. This role requires a professional or Master’s level qualification. There are also, Registered Nurses who can provide clinical assessment, review functional needs and consider reasonable adjustments to the court, a Consultant Forensic Psychiatrist for making clinical recommendations, reviewing fitness to plead and ability to effectively participate in the court process and a clinical or forensic psychologist to provide reports for the court and to conduct assessments.

During these reviews, the records available to assessing clinicians included past and current clinical and custody records, Person Escort Records (PER: which detail the risk of self-harm of those being transferred to court) and information from referral sources, including solicitors and other healthcare staff. Diagnoses were assigned by assessing clinicians after reviewing historical clinical records and undertaking new clinical assessments.

### Analysis

Data were analysed using the Statistical Package for Social Sciences (SPSS 25), using descriptive statistics, chi-square tests and measures of association.

### Ethical considerations

These data were routinely collected and aggregated by NHS England (NHSE), as part of their routine management and reporting of trends within these services. Administrative permissions to use the current data were given from both NHSE and Together who provide criminal justice services at the courts and are responsible for generating and managing activity data. All data were anonymised before being sent to the research team.

## Results

### Participants

Of the 9088 defendants referred to the CMHLDS, 79.1% (*n* = 7186) were male and 18.9%, (*n* = 1719) were female, with 2% (*n* = 183) unknown. Age for all defendants ranged from 17 to 89 years (M = 37.5, SD = 11.26). The most common age group was between 30–39 years old. The ethnicity of defendants was White 50.2% (*n* = 4566), Black 20.2% (*n* = 1832), Asian 12%, (*n* = 1088), Mixed ethnicity 6.1% (*n* = 558) and Chinese 0.2% (*n* = 16). The remainder were recorded as undeclared or unknown 6.4% (*n* = 580) or as any other ethnic group at 4.9%, (*n* = 448). Data was not recorded for schizophrenia (or other delusional disorder) (SDD), Bipolar affective disorder (BPAD), Anxiety, depression and NDDs in 183 cases, for personality disorder (PD) in 205 cases, for substance use disorder (SUD), in 2605 cases and for alcohol use disorder (AUD) in 2649 cases.

### Psychiatric disorder – rates, gender & ethnicity

An identifiable psychiatric disorder was present in 72.8% (*n* = 6616) of defendants. The highest rates were for SDD 23.2% (*n* = 2110), PD 19.8% (*n* = 1739) and depression 19.3% (*n* = 1720). Males had significantly higher rates of SDD and NDD, whereas females had significantly higher rates of PD, anxiety depression and BPAD. Significantly higher rates of schizophrenia were found in black defendants, who had rates twice as high as white defendants, and people of white ethnicity had the highest rates of depression and PD. (Please see Tables 1 and 2).

**Table 1:** Rates of mental disorder of court defendants by gender

	Male - %, (*n* = 7186) unless stated	Female- %, (*n* = 1719) unless stated	Chi-square * = significant at <005 level	Total (*n* = 8905)
Schizophrenia or other delusional dis.	25.0%, (1799)	17.0%, (293)	X2= (1), 8905, 49.271 *P* < .001***	23.5%, (2092)
Depression	18.2%, (1305)	24.1%, (415)	X2= (1), 8905, 31.848, *P* < .001***	19.3%, (1720)
Bipolar Affective disorder	4.4%, (315)	7.4%, (127)	X2= (1), 8905, 26.546, *P* < .001***	5%, (442)
Anxiety	9.1%, (651)	11.1%, (191)	X2= (1), 8905, 6.821, *P* < .009**	9.5%, (842)
Neurodevelopmental Disorders	6.0%, (433)	4.0%, (68)	X2= (1).8783,11.193 *P* = <.001***	5.6% (501)
Personality Disorder	(*n* = 7086)	(*n* = 1697)		(*n* = 1719)
	17.9%, (1269)	27.7%, (470)	X2= (1), 8905, 85.592, *P* < .001***	19.8%, (1739)
Substance Misuse	(*n* = 5231)	(*n* = 1252)		(*n* = 6483)
	44.3%, (2315)	39.0%, (488)	X2= (7), 6483, 11.466 *P* = <.001***	43.2%, (2803)
Alcohol Misuse	(*n* = 5198)	(*n* = 1242)		(*n* = 6439)
	23.3%, (1672)	25.2%, (434)	X2= (7), 6439, 3.592, *P* = <.166	32.7%, (2106)

**p<.01,  ***p<.001

**Table 2:** Rates of mental disorder of court defendants by ethnicity

	White - %, (*n* = 4566) unless stated	Mixed- %, (*n* = 558) unless stated	Asian-%, (*n* = 1088)	Black- %, (*n* = 1832)	Chinese- %, (*n* = 16)	Other ethnic group -%, (n) = 448	Chi-square * = significant at <005 level
Schizophrenia or other delusional dis.	17.1%, (782)	28.9%, (161)	29.0%, (315)	37.2%, (681)	18.8%, (3)	21.6%, (97)	X2= (5), 8508, 316.274 *P* < 001***
Depression	22.1%, (1007)	16.5%, (92)	21.1%, (230)	15.3%, (281)	12.5%, (2)	15.2%, (68)	X2= (5), 8508, 49.311 *P* P < .001***
Bipolar Affective disorder	5.2%, (237)	6.5%, (36)	3.6%, (39)	5.0%, (91)	0.0%, (0)	5.6%, (25)	X2= (5), 8508, 8.511 *P* < ..130
Anxiety	10.8% (491)	10.4%, (58)	10.2%, (111)	7.2%, (132)	6.3%, (1)	6.9%, (31)	X2= (5), 8508, 23.624 *P* < .001***
Neurodevelopmental Disorders	6.5%, (297)	6.8%, (38)	3.6%, (90)	4.9%, (90)	0.0%, (0)	4.0%, (18)	X2= (5), 8508, 21.378 *P* < .001***
Personality Disorder	(*n* = 4512)	(*n* = 553)	(*n* = 1062)	(*n* = 1803)		(*n* = 443)	
	21.1% (952)	18.1%, (435)	15.2%, (227)	12.6%, (227)	12.5%, (2)	19.2%, (25)	X2= (5), 8309, 70.818 *P* < .001***
Substance Misuse	(*n* = 3383)	(*n* = 558)	(*n* = 1088)	(*n* = 1088)	(*n* = 13)	(*n* = 310)	
	43.7%, (1479)	53.3%, (232)	36.4%, (610)	45.6%, (610)	15.4%, (2)	35.5%, (110)	X2= (5), 6317, 49.388 *P* < .001***
Alcohol Misuse	(*n* = 3361)	(*n* = 434)	(*n* = 1088)	(*n* = 1088)	(*n* = 14)	(*n* = 1312)	
	29.3%, (1340)	19.9%, (111)	22.1%, (331)	18.1%, (331)	6.3%, (1)	13.2%, (59)	X2= (5), 6278, 172.876 *P* < .001***

**p<.01, ***p<.001

### Alcohol and substance misuse disorders - rates, gender & ethnicity

SUD was recorded at a higher rate, 43.2%, (*n* = 2803), than alcohol misuse 32.7% (*n* = 2106), among defendants. AUD was higher amongst females 25.2% (*n* = 434) than males (23.3%; *n* = 1672)) although not reaching significance, whilst substance misuse rates were significantly higher in males at 44.3% (*n* = 2315) than females (39%; *n* = 488. Significantly high rates of substance and alcohol misuse comorbidity were found in defendants presenting with the following diagnoses, schizophrenia, anxiety disorders and personality disorders. A significant association was also seen between alcohol use and depression (see Table 3). People of mixed ethnicity had the highest rates of SUD 232, (53.3%), whereas people of white ethnicity were the most likely to have AUD 1340, (39.9%.).

**Table 3:** Association of comorbid alcohol and substance misuse with common mental and neurodevelopmental disorders

	Substance Use Dx, n, (%)	Pearson’s Chi square (2 sided) * = significant	Alcohol Use Dx, n, (%)	Pearson’s Chi square (2 sided) * = significant
Schizophrenia or other delusional dis.	805, (45.8%)	X2, (1), 6499, 6.294, *P* < .012*	438, (20.7%)	X2, (1), 6499, 63.599, *P* < .001***
Depression	645, (41.6%)	X2, (1), 6499, 2.239 *P* < .135	601, (28.5%)	X2, (1), 6499, 35.856, *P* < .001***
Bipolar Affective Disorder	152, (31.4%)	X2, (1), 6499,.552, *P* < .457	111, (5.3%)	X2, (1), 6499, 1.020, *P* < .313
Anxiety	289, (38.2%)	X2, (1), 6499, 9.101, *P* < .003*	286, (13.5%)	X2, (1), 6499, 9.211, *P*<,.002**
Personality Disorders	517, (48.6%)	X2, (1), 6297, 15.219, *P* < .001*	408, (19.7%)	X2, (1), 6435, 20.836, *P* < .001*
Neurodevelopmental Disorders	173, (41,5%)	X2, (1), 6499, .586, *P* < .444	136, (6.4%)	X2, (1), 6449, 20.836, *P* < .846

**p<.01, ***p<.001

### Neurodevelopmental Disorders (NDDs) – rates, gender & ethnicity

NDDs were present in 5.5% (*n* = 503) of defendants. Gender data were available for 460 cases, and 86.7%, (*n* = 399) were male, while 13.3% (*n* = 61) were female. The most common NDD was ID, comprising 3.8% (*n* = 341) of the sample, followed by ADHD 1.2% (*n* = 110) and ASD 1.1% (*n* = 100) respectively. Fifty-four (10.7%) of the group with NDDs presented with more than one NDD, the most common combination was ASD and ID (6.5%: *n* = 32). People of white and mixed ethnicity were the more likely to have a NDD diagnosis, whereas people of Asian ethnicity were the least likely to be diagnosed with a NDD. (Please see Tables 1 and 2).

## Discussion

Evaluations of CMHLDS indicate that they are effective in identifying offenders with psychiatric disorders [21], however, there is little research to say how many defendants with a mental health need entering court remain unidentified and for who referral would be beneficial. There is a need for more research to understand what services work best, in which circumstances, and how best to cater for people who present in vulnerable, or so-called marginalised, groups [1, 19, 22, 23], Information from screening within Police Mental Health Liaison and Diversion services (PMHLDS) and CMHLDS for mental disorders may provide early identification of vulnerabilities, as well as highlighting potential future vulnerabilities. This study of 9088 defendants across five CMHLDS in London is one of the first to examine such a large number of defendants and identify the nature of mental disorder and demographic characteristics of those entering CMHLDS, and the results demonstrate very high rates of psychiatric morbidity compared to community samples.

### Characteristics of the defendants

Males in the current study comprised the majority of defendants 79.1% (*n* = 7186), consistent with 79.1% (163) reported by a Cornish Police MHLDS [3, 24] and slightly lower than the 79.5% (1778) reported from an Australian mental health court. The ethnic representation of the sample reflected the diversity of London. Black (20.2%) and mixed ethnicity defendants (6.1%) were overrepresented in line with the Lammy review and found at higher rates, compared to the London population rates of 13.3 and 5% respectively [25]. The current study identified 72.8% with a psychiatric disorder, this is consistent with 71% from the national evaluation [11]. The remaining referrals are made up of other vulnerable groups previously mentioned and those referred where following assessment there was no evidence of a psychiatric disorder. Female defendants had high recorded rates of depression and PD. This study has also identified the over-representation of severe mental illness for specific ethnic groups, including higher rates of schizophrenia in Black, Asian and mixed ethnic groups, consistent with previous studies [26]. Further research is necessary to fully understand whether this phenomenon is isolated to these services in London or applicable more widely within the criminal justice system in England and Wales, the wider United Kingdom, and internationally. Female defendants comprised just under a fifth of the group which is a high figure compared to other studies that have recorded rates of females at 5% [19] in line with the female prison population [27]. and requires further research and scrutiny of local practices and operational guidance.

### Rates of psychiatric disorder

Although CMHLDS are meant to provide a model that caters [28] for all illnesses and vulnerabilities, the data indicates that the focus remains on severe mental illness (SMI), while other common psychiatric disorders, including depression, anxiety and Post Traumatic Stress Disorder, are not assessed to the same extent which has been found in another recent study [29]. In this study, schizophrenia and delusional disorders were recorded at rates of, 23.2%, PD 19.2% depression 19% and anxiety 9.3%, which were seen at much higher rates than in community samples [30]. The available literature shows considerable variation between individual schemes. However, there is little consistency in the reported rates between liaison and diversion services. For example, a previous study of 206 detainees in a police mental health liaison diversion service (PMHLDS) from Cornwall, UK reported rates of anxiety and/or depression of 36.7% (*n* = 81) which is comparable to this study, Psychotic disorders including BPAD of 16.3% (*n* = 36) which is slightly lower than this study but much lower rates of PD at 8.6% (*n* = 19) compared to this study with a rate of 8.1% (*n* = 18) for ADHD so slightly higher than this London study [3, 20]. Another study of 1858 attendees in Newcastle, Australia, reported rates as follows: BPAD 6.0%, Depression 11.9%, and PD 7.1% which are much lower rates than this study but a comparable rate of Psychotic disorder of 17.0% [24]. These variations may arise for several different reasons, including location and type of liaison and diversion service (e.g., rural or urban, police or court), and different policies and referral pathways operating within different legal jurisdictions. Variations in prevalence rates may reflect differences between the definition and severity of presentations, how cases are screened, and differences in the incorporation of historical clinical records [31]. Nevertheless, our study confirms high rates of severe mental illness, in keeping with the wider literature, but it also reports higher rates of PD than have been found elsewhere which requires further exploration.

### Rates of substance and alcohol use

The study found high rates of substance 43.2%, (2803) and alcohol misuse 32.7%, (2106) amongst defendants. This is likely to be an underestimate given the large number of cases where this data was not recorded. This compares to systematic review findings of recently incarcerated men and women with SUD, which reported a pooled prevalence of 30% (range 10–61%) and AUD of 24% (range 16–51%) [29]. From the Danish psychiatric population registers (*N* = 463,003), a SUD lifetime prevalence of 30.4% (140, 811) was reported. This compares to a rate of 9.4%; (3.2 million) for adults aged 16–59 years old within a community sample, with the highest rates between the ages of 16 to 24 years old of 21%; (1.3 million), who had taken a drug in the last year [32]. Comparison can be difficult due to a range of definitions and thresholds to measure potential harm related to substance and alcohol use. Given the widespread use of alcohol and drugs, related statistics of population habits can still highlight the scale of AUD and SUD in both psychiatric and criminal justice populations, compared to the general society. For example, alcohol consumption, exceeding the weekly limit of 14 units of alcohol has been reported in 30% of men in 2019 in the UK, this is twice as high as the percentage of women [31]. The 12-month prevalence of AUD in the USA is 13.9%. In the UK the rates of alcohol dependence range between 0.64–3.5 per 100 across local authorities [33].

### Rates of Neurodevelopmental disorders (NDDs)

Rates of NDDs are often affected by a lack of screening programmes, and legal and diagnostic thresholds [34]. Nevertheless, it is important to identify people with these disorders as they progress through the criminal justice system to ensure they receive the care and treatment they need, and also to ensure their right to a fair trial by signalling cases in which additional support is required, or where having the capacity to participate may be an issue [35, 36]. Although the literature regarding mental disorders within police and CMHLDS is improving, there is still a paucity of literature regarding NDDs in these settings, despite evidence of high rates of mental health comorbidity and increased vulnerability amongst people screening positive for NDDs [34, 37]. In PMHLDS rates of specific NDDs have been reported at differing levels, which may be accounted for by the variation in methods, and quality between studies. There are studies consistent with rates in community samples and similar to findings in this study, including a study in the North East of England that reported 3.6% with ID and 1.2% with autism spectrum disorder [38]. A study in a South London Police station involving a cohort of 134 people found a rate of 11% for ADHD and 4% for ID [17]. However, over time as identification improves there is some evidence of a drop in figures of the numbers of people with intellectual disability identified in liaison and diversion services with one study reporting figures for ID at 5.0% of cases in 1991/1992, dropping to 1.2% in 2015/2016 [29]. Although these figures need to be interpreted with caution as this was the trend for other disorders reported, apart from mood and personality disorders which were seen at higher rates. Although the quality of these studies varies, the number of defendants with NDDs is sufficient to require a specific approach within CMHLDS for this group that is not the same as the generic offer made to those who present with severe mental disorders.

It is anticipated given the lack of expertise in the identification of NDDs currently within liaison and diversion services which were traditionally developed for severe mental illness that over time as services become more aware of other vulnerabilities, there will be an improvement in the identification of defendants with NDDs. There is now good evidence to say that the numbers of defendants with NDDs are sufficient to require a specific approach that is not the same as those who present with severe and common mental disorders. We need to understand what that model should look like by further evaluation on how we can successfully integrate practitioners with the expertise of NDDs into existing police and court services [1].

To date, there are few studies examining the characteristics of a large number of defendants referred to CMHLDS, so this study as well as increasing our understanding of this group in terms of planning future research also offers a comparison with other liaison and diversion models internationally in terms of who they assess and outcomes achieved. This study also informs the debate due to the level of need identified on whether services should be enhanced or whether some groups warrant having specific service provisions, such as substance and alcohol use or NDDs.

### Limitations

This study is limited to an evaluation of data that is routinely collected as part of the operations of liaison and diversion services, and although not designed as a prevalence study, it is one of the largest studies to look at the nature of mental disorders in defendants seen by CMHLDS. One potential criticism of large service data sets is incomplete data entry, which is a possibility in this case, and we have therefore acknowledged this where it has occurred. There are several reasons why this can happen, including time pressures, clinical prioritisation, and lack of expertise in data management. As with all clinical records it could not be guaranteed that these records were available in every case.

In terms of informing future service development, there is a risk that activity and use may not be stable over time and could be influenced by factors such as changes in policy. However, the demographic characteristics examined over the two years of the study are consistent with previous estimates. Trends however are largely unavailable as data on some groups such as those with NDD has not been reported so frequently.

Another limitation of this study is reliance upon existing records and clinical assessments to assign diagnoses, and we acknowledge that diagnostic instruments were not utilised as a part of the research design. It is also generally recognised that electronic health record systems may not be primarily designed for the sharing of information between agencies, and they may be hampered by a lack of guidance as regards the overall recording of clinical information [38, 39].

## Conclusions

This study may be the largest of its kind and has improved our understanding of the characteristics of defendants entering CMHLDS. It has demonstrated a clear need for CMHLDS that have expertise working with a highly morbid population, with high levels of complexity and with multiple needs. This defendant group presents with very high levels of mental disorder and there were variations between ethnic groups as regards reported prevalence with some ethnic groups presenting with disproportionately high levels of these disorders. Alcohol and substance misuse are also very common and can act as significant complicating factors in clinical presentation. Given these results, service designs will need to incorporate effective screening to identify a range of disorders and provide sufficient resources to manage healthcare pathways and divert people into arrangements for care where appropriate. More targeted research is required to consider outcomes relating to diversion to health and social care services, or community orders, and understand how effective use of resources within police stations and courts can improve health outcomes for the wider group of vulnerable defendants. It will be important to ensure that protected characteristics for example ethnicity, race, gender and age do not introduce further barriers to accessing appropriate healthcare.

## Data Availability

The datasets generated and/or analysed during the current study are not publicly available due to being the property of the NHS for service evaluation and comparison. Data cleaned and anonymised that was used in the study can be available from the corresponding author on reasonable request and agreement of the original owners.

## Footnotes

1 Magistrates’ courts are the lower courts in England and Wales. 152,758 cases were disposed, with 123,023 trials in 2018 the lowest in the series (2003–2018). (MoJ, 2019) https://assets.publishing.service.gov.uk/government/uploads/system/uploads/attachment_data/file/812556/ccsq-bulletin-q1-2019.pdf

## References

[1] Chaplin E, McCarthy J, Marshall-Tate K, Ali S, Xenitidis K, Childs J, et al. Evaluation of a liaison and diversion Court Mental Health Service for defendants with neurodevelopmental disorders. Res Dev Disabil. 2021;119:104103. 10.1016/j.ridd.2021.104103. Epub 2021 Oct 7. PMID: 34628339. 10.1016/j.ridd.2021.104103 34628339

[2] Disley E, Taylor C, Kruithof K, Winpenny E, Liddle M, Sutherland A, Lilford R, Wright S, McAteer L, Francis V (2016). Evaluation of the offender liaison and diversion trial schemes.

[3] Earl F, Cocksedge K, Morgan J, Bolt M (2017). Evaluating liaison and diversion schemes: an analysis of health, criminal and economic data. J Forensic Psychiatry Psychol.

[4] Birmingham L, Awonogun O, Ryland H (2017). Diversion from custody: An update. BJPsych Adv.

[5] Forrester A, Hopkin G (2019). Mental health in the criminal justice system: a pathways approach to service and research design. Crim Behav Ment Health.

[6] Criminal Justice Joint Inspections England and Wales. A joint thematic inspection of the criminal justice journey for individuals with mental health needs and disorders. 2021. https://www.justiceinspectorates.gov.uk/hmicfrs/publications/joint-thematic-inspection-criminal-justice-mentalhealth-needs/.

[7] Brooker C, Coid J. Mental health services are failing the criminal justice system. BMJ. 2022;376. 10.1136/bmj-2021-069776 35082163

[8] Shaw J, Creed F, Price J, Huxley P, Tomenson B (1999). Prevalence and detection of serious psychiatric disorder in defendants attending court. Lancet.

[9] Corston J. The Corston report: a report by Baroness Jean Corston of a review of women with particular vulnerabilities in the criminal justice system: the need for a distinct, radically different, visibly-led, strategic, proportionate, holistic, woman-centred, integrated approach: Home Office; 2007. https://www.asdan.org.uk/media/ek3p22qw/corston-report-march-2007.pdf.

[10] Bradley KJCB (2009). The Bradley Report: Lord Bradley's review of people with mental health problems or learning disabilities in the criminal justice system: Department of Health London.

[11] Disley E, Gkousis E, Hulme S, Morley KI, Pollard J, Saunders CL (2021). Outcome Evaluation of the National Model for Liaison and Diversion.

[12] Mental health [Internet]. Our World In Data. 2020 [cited 01/05.2020]. Available from: 'https://ourworldindata.org/mental-health.

[13] Alcohol consumption [Internet]. Our World In Data. 2020 [cited 01/05.2020]. Available from: https://ourworldindata.org/alcohol-consumption.

[14] Neurodevelopmental Disorders [Internet]. Our World In Data. 2020 [cited 01/05.2020]. Available from: 'https://ourworldindata.org/mental-health.

[15] McKinnon I, Srivastava S, Kaler G, Grubin D (2013). Screening for psychiatric morbidity in police custody: results from the HELP-PC project. Psychiatrist.

[16] Forrester A, Samele C, Slade K, Craig T, Valmaggia L (2017). Demographic and clinical characteristics of 1092 consecutive police custody mental health referrals. J Forensic Psychiatry Psychol.

[17] Samele C, McKinnon I, Brown P, Srivastava S, Arnold A, Hallett N (2021). The prevalence of mental illness and unmet needs of police custody detainees. Crim Behav Ment Health.

[18] Forrester A, Samele C, Slade K, Craig T, Valmaggia L (2016). Suicide ideation amongst people referred for mental health assessment in police custody. J Crim Psychol.

[19] Forrester A, Hopkin G, Bryant L, Slade K, Samele C (2020). Alternatives to custodial remand for women in the criminal justice system: A multi-sector approach. Crim Behav Ment Health..

[20] Lammy D. The Lammy Review: An Independent Review into the Treatment of, and Outcomes for, Black, Asian and Minority Ethnic Individuals in the Criminal Justice System. London; 2017. https://assets.publishing.service.gov.uk/government/uploads/system/uploads/attachment_data/file/643001/lammy-review-final-report.pdf.

[21] Scott DA, McGilloway S, Dempster M, Browne F, Donnelly M (2013). Effectiveness of criminal justice liaison and diversion services for offenders with mental disorders: a review. Psychiatr Serv.

[22] Srivastava S, Forrester A, Davies S, Nadkarni R (2013). Developing criminal justice liaison and diversion services: research priorities and international learning. Crim Behav Mental Health..

[23] Hopkin G, Chaplin L, Slade K, Craster L, Valmaggia L, Samele C (2020). Differences between homeless and non-homeless people in a matched sample referred for mental health reasons in police custody. Int J Soc Psychiatry.

[24] Sly KA, Sharples J, Lewin TJ, Bench CJ (2009). Court outcomes for clients referred to a community mental health court liaison service. Int J Law Psychiatry.

[25] UK Government. Gov UK Criminal court statistics quarterly: January to March 2019 https://www.gov.uk/government/statistics/criminal-court-statistics-quarterly-january-to-march-2019. 2019.

[26] Maura J, Weisman de Mamani A (2017). Mental Health Disparities, Treatment Engagement, and Attrition Among Racial/Ethnic Minorities with Severe Mental Illness: A Review. J Clin Psychol Med Settings.

[27] Ministry of Justice. Statistics on Women and the Criminal Justice System 2019-A Ministry of Justice publication under Section 95 of the Criminal Justice Act 1991. 2020. https://assets.publishing.service.gov.uk/government/uploads/system/uploads/attachment_data/file/938360/statistics-on-women-and-the-criminal-justice-system-2019.pdf.

[28] NHS England. Liaison and diversion standard service specification. London: The National Liaison and Diversion Programme, NHS England and Improvement; 2019. https://www.england.nhs.uk/wp-content/uploads/2019/12/national-liaison-and-diversion-service-specification-2019.pdf.

[29] Ryland H, Forrester A, Exworthy T, Gallagher S, Ramsay L, Khan AA (2021). Liaison and diversion services in South East London: Referral patterns over a 25-year period. Med Leg J..

[30] King M, Nazareth I, Levy G, Walker C, Morris R, Weich S (2008). Prevalence of common mental disorders in general practice attendees across Europe. Br J Psychiatry.

[31] Cooper S, Smiley E, Morrison J, Allan L, Williamson A (2007). Prevalence of and associations with mental ill-health in adults with intellectual disabilities. Br J Psychiatry.

[32] Office of National Statistics. Drug misuse in England and Wales: year ending March 2020: An overview of the extent and trends of illicit drug use for the year ending March 2020. Data are from the Crime Survey for England and Wales 2020. https://www.ons.gov.uk/peoplepopulationandcommunity/crimeandjustice/articles/drugmisuseinenglandandwales/ingmarch2020.

[33] House of Commons Library. Alcohol statistics England 2021. https://researchbriefings.files.parliament.uk/documents/CBP-7626/CBP-7626.pdf.

[34] McCarthy J, Chaplin E, Forrester A, Underwood L, Hayward H, Sabet J (2019). Prisoners with neurodevelopmental difficulties: Vulnerabilities for mental illness and self-harm. Crim Behav Ment Health.

[35] Brown P (2019). Unfitness to plead in England and Wales: Historical development and contemporary dilemmas. Med Sci Law.

[36] Brown P (2019). Modernising fitness to plead. Med Sci Law.

[37] McCarthy J, Chaplin E, Underwood L, Forrester A, Hayward H, Sabet J (2016). Characteristics of prisoners with neurodevelopmental disorders and difficulties. J Intellect Disabil Res.

[38] McKenna D, Murphy H, Rosenbrier C, Soulsby A, Lyall A, Keown P (2019). Referrals to a mental health criminal justice Liaison and diversion team in the North East of England. J Forensic Psychiatry Psychol.

[39] Shah AD, Quinn NJ, Chaudhry A, Sullivan R, Costello J, O'Riordan D, et al. Recording problems and diagnoses in clinical care: developing guidance for healthcare professionals and system designers. BMJ Health Care Inform. 2019;26(1). 10.1136/bmjhci-2019-100106 PMC7062352 31874855

